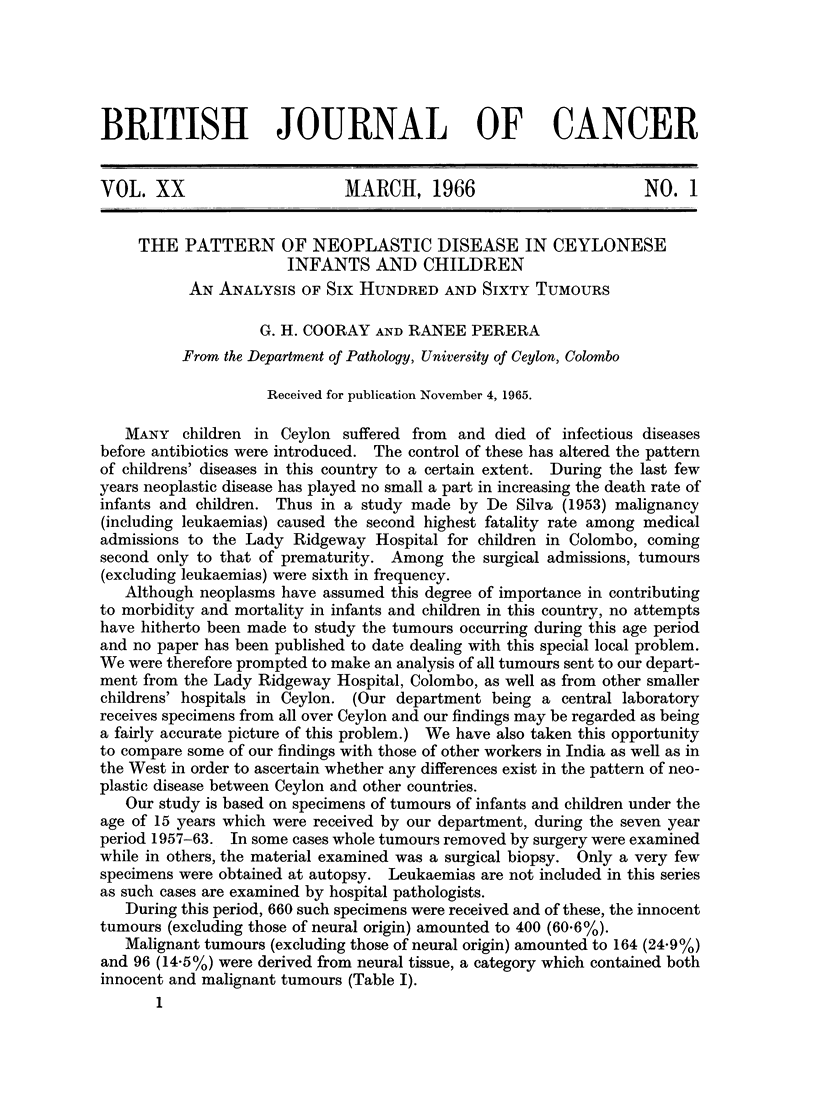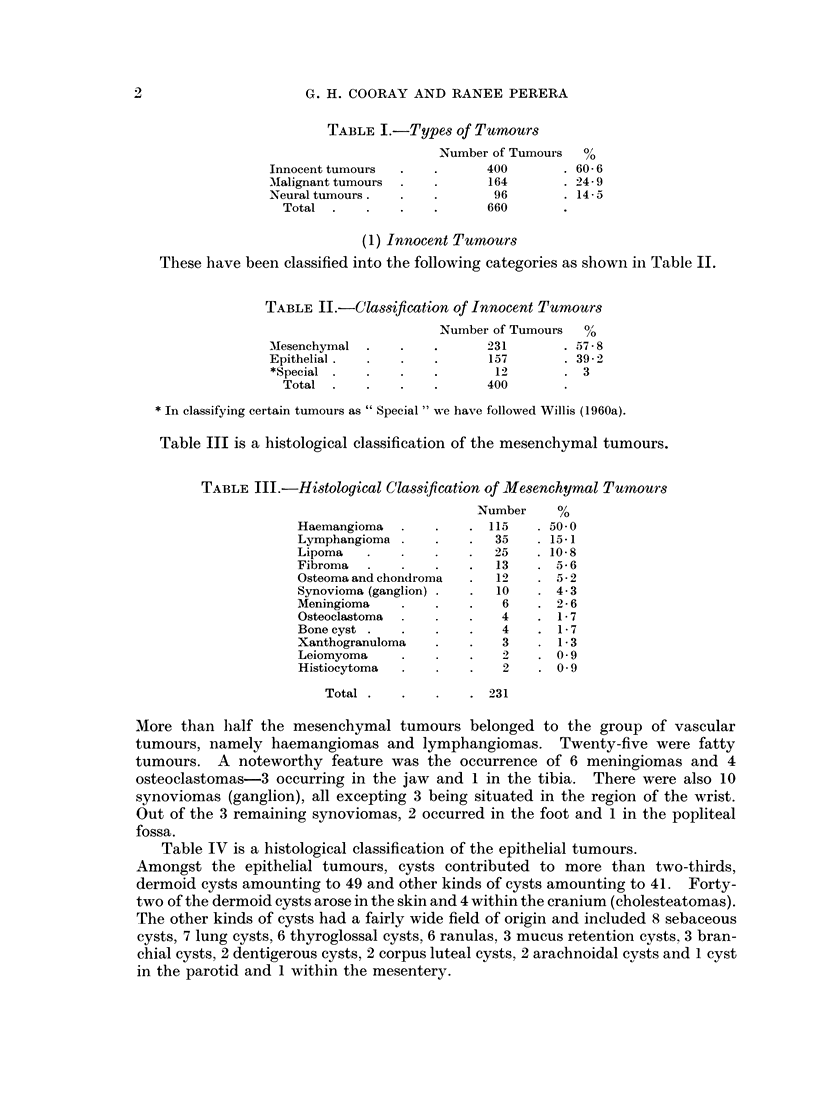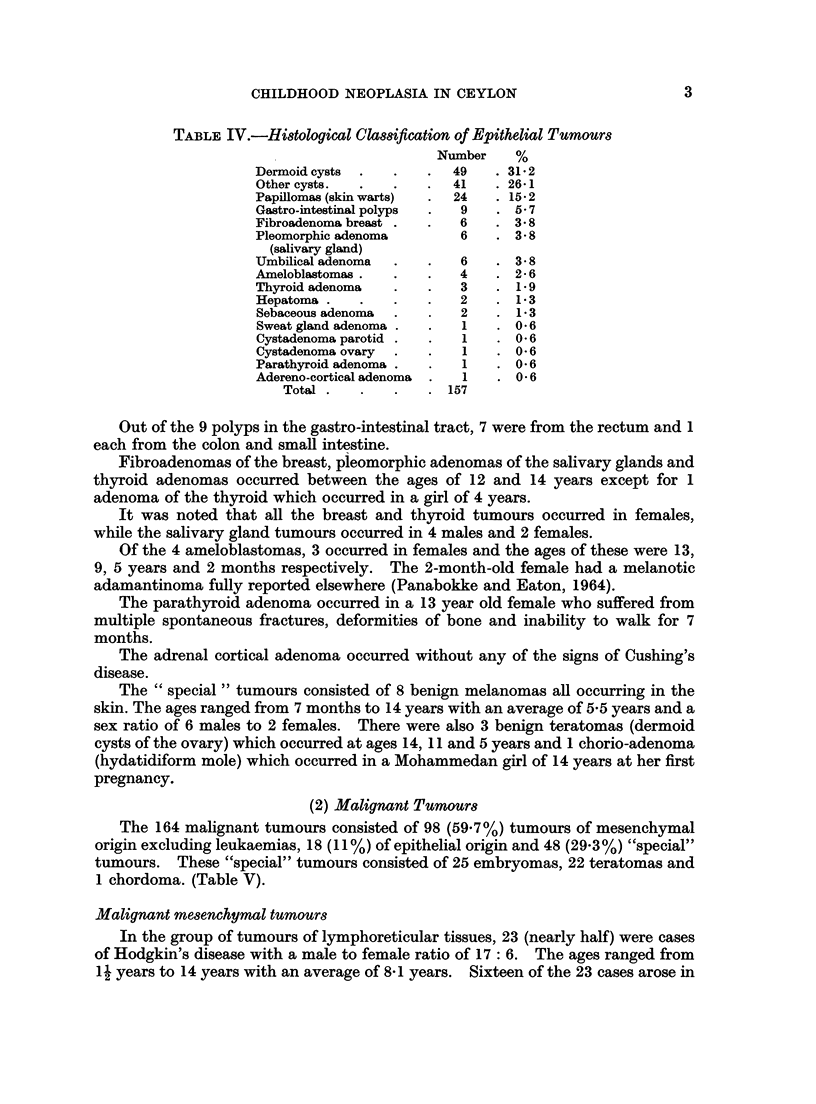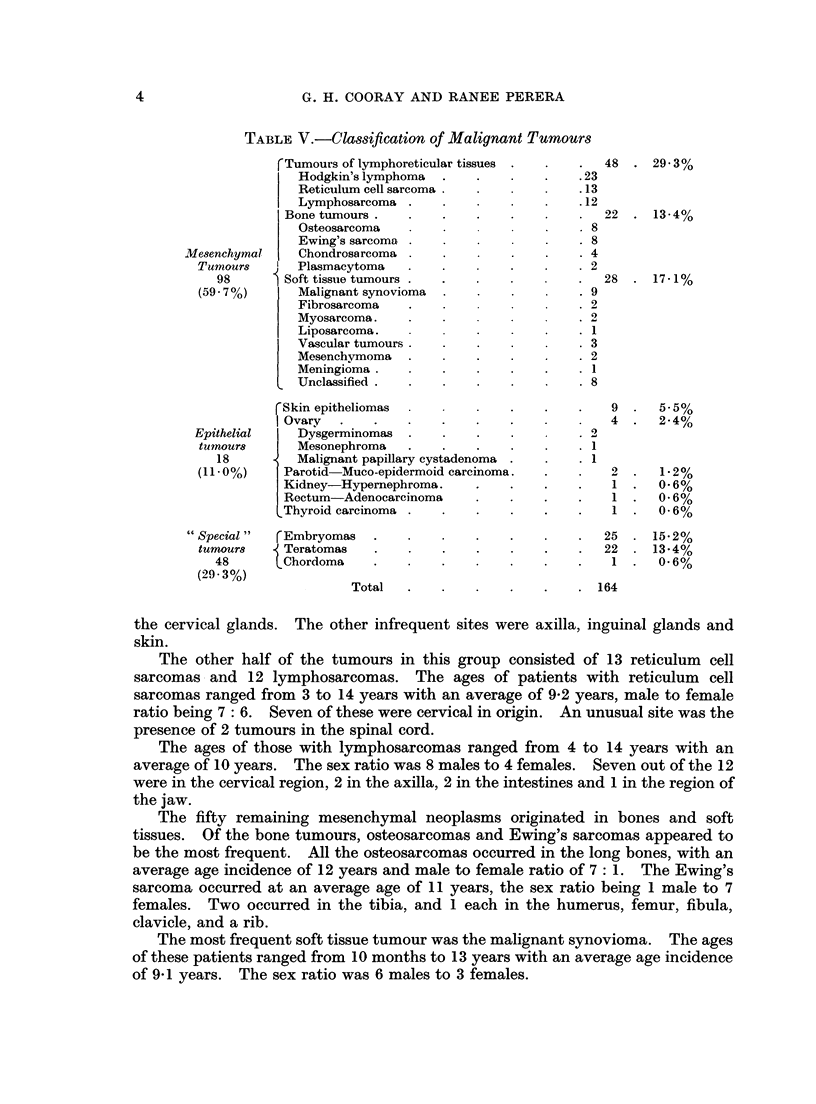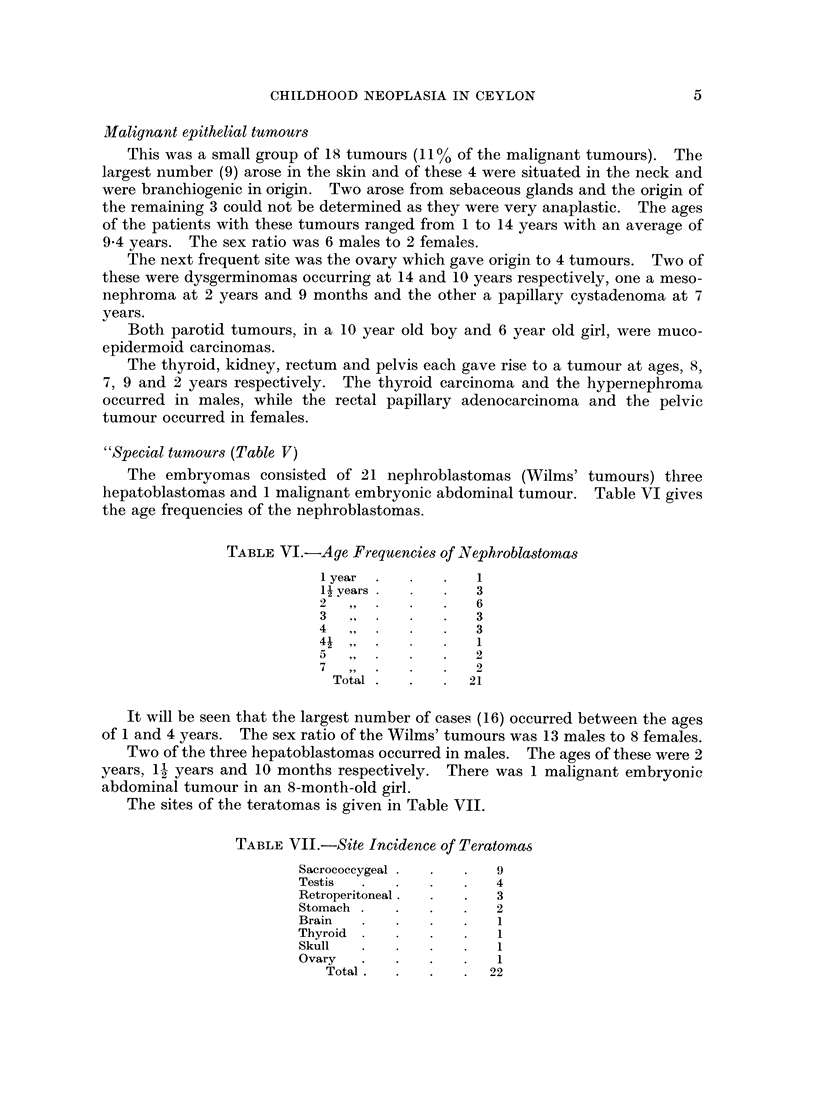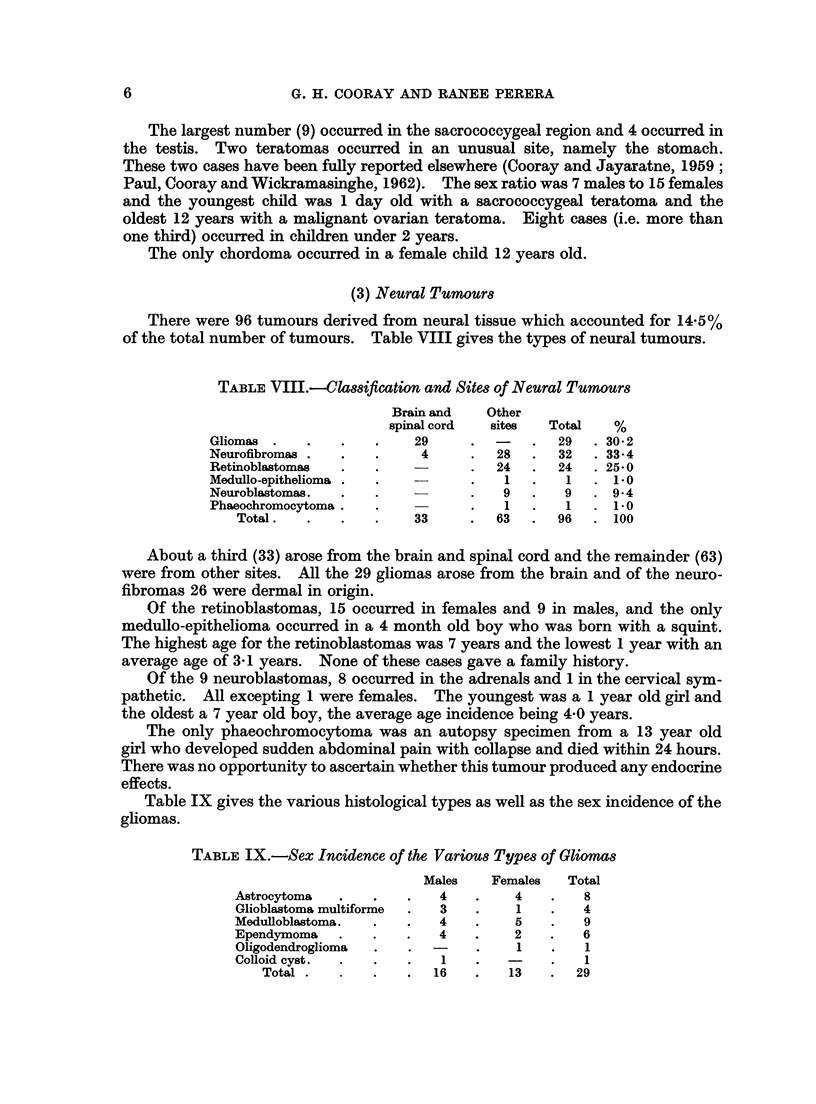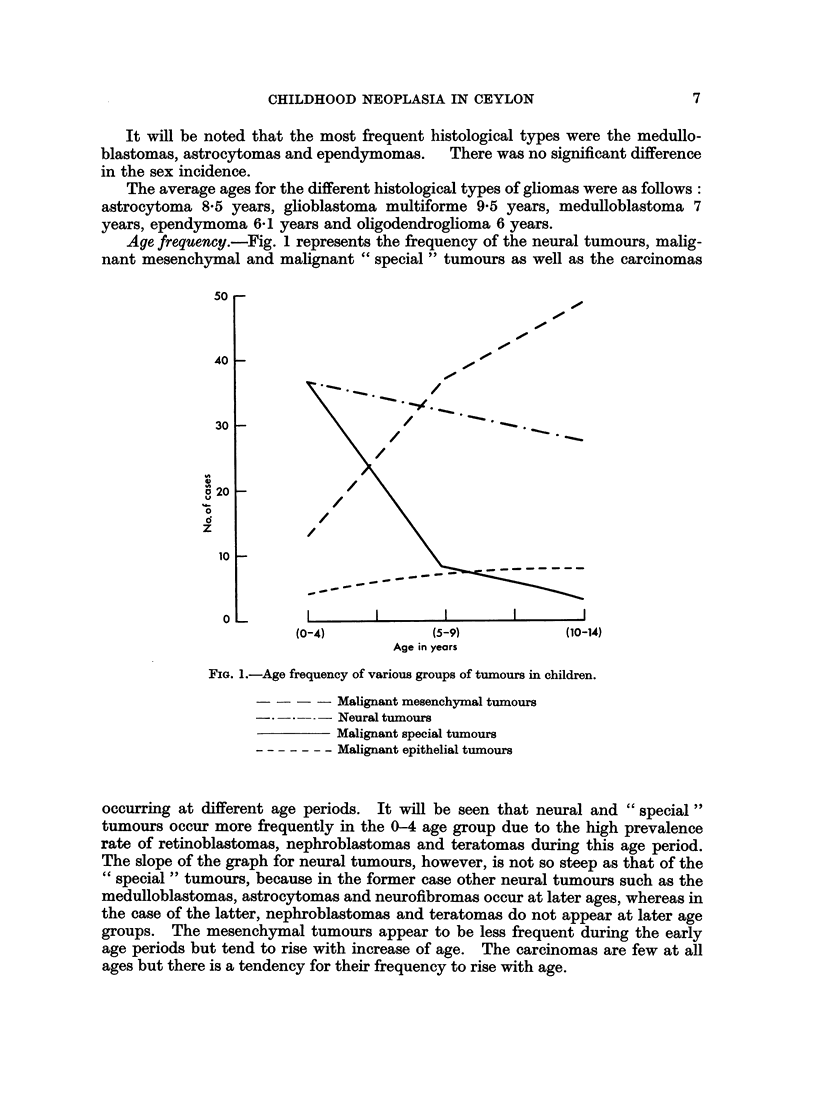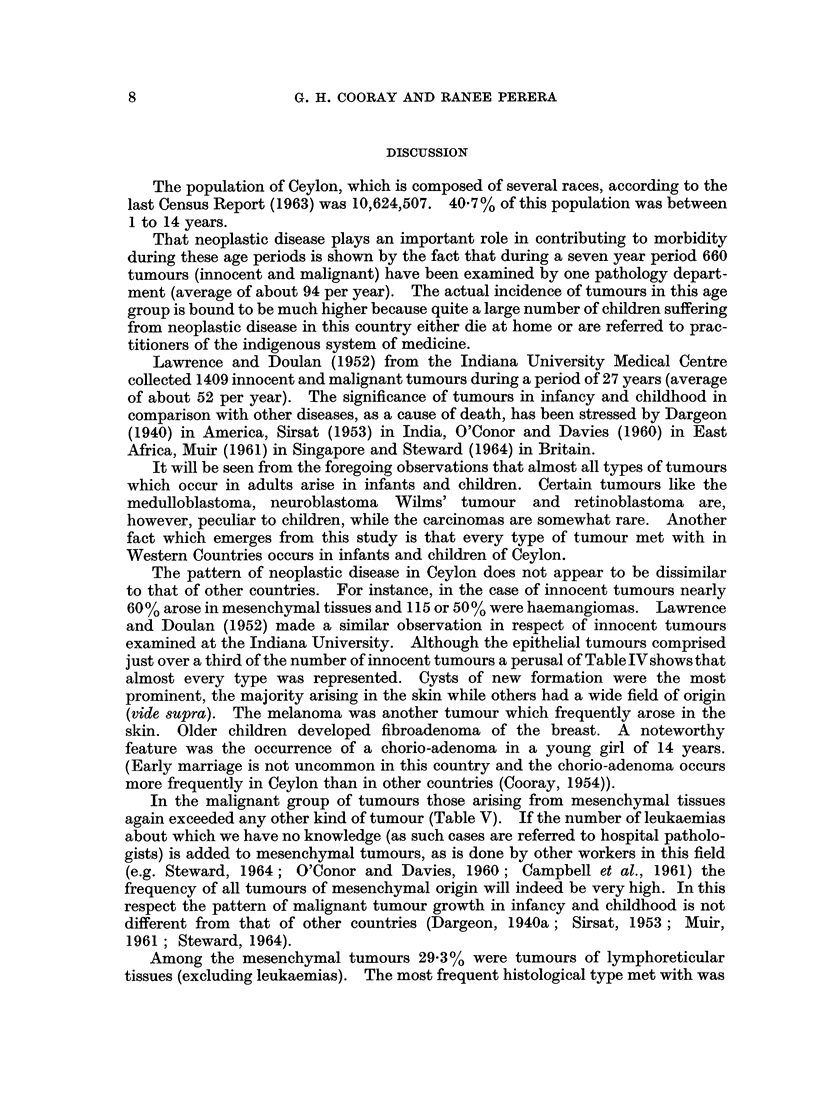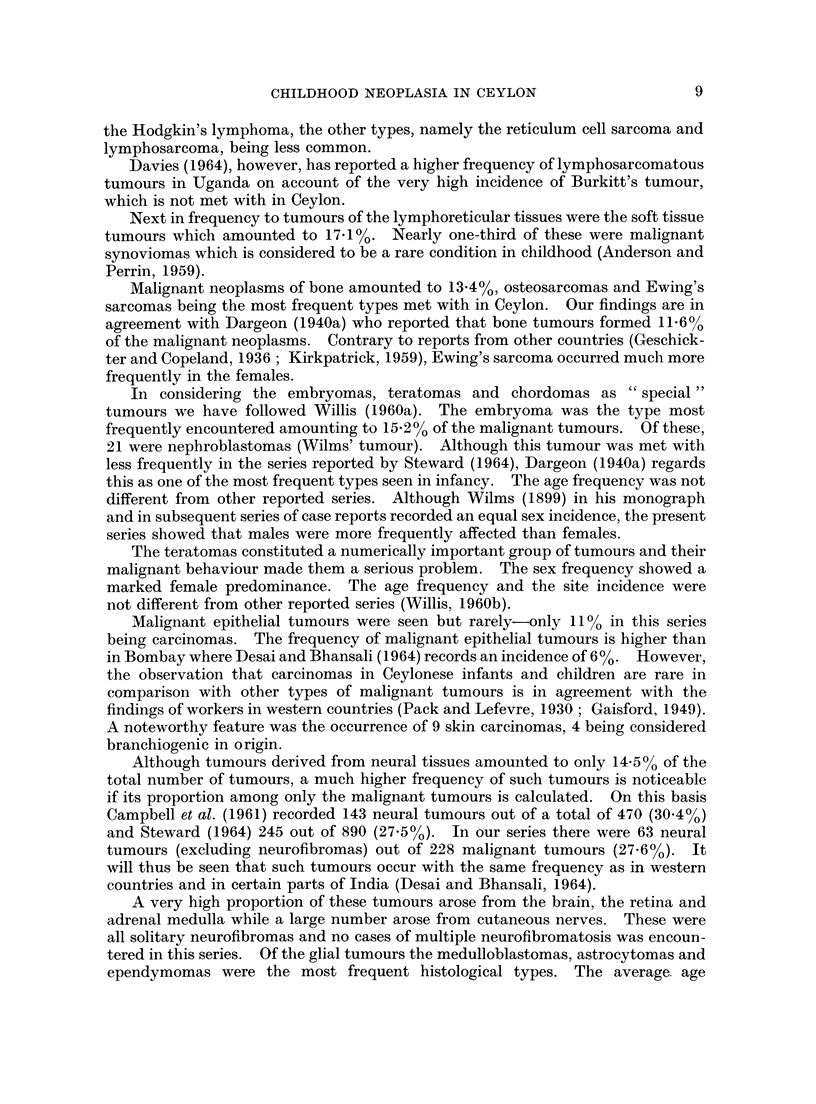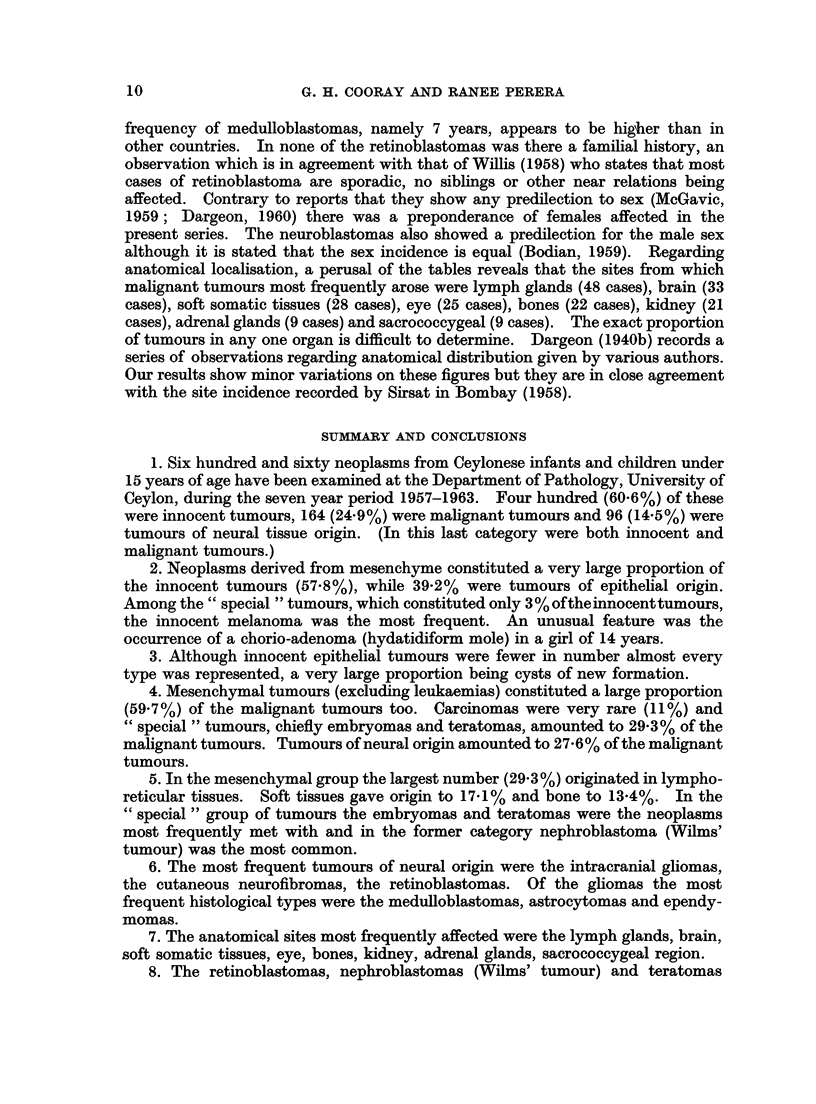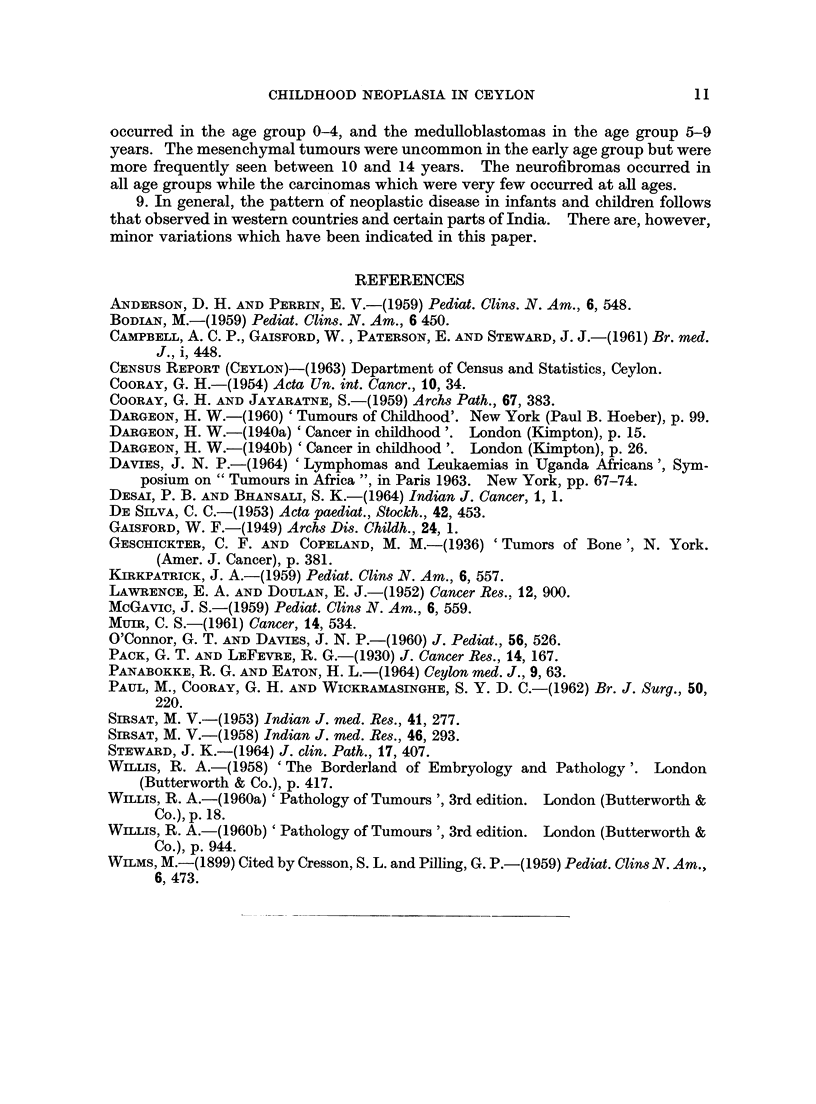# The Pattern of Neoplastic Disease in Ceylonese Infants and Children

**Published:** 1966-03

**Authors:** G. H. Cooray, Ranee Perera


BRITISH JOURNAL OF CANCER

VOL. XX MARCH, 1966 NO. 1

### THE PATTERN OF NEOPLASTIC DISEASE IN CEYLONESE INFANTS AND CHILDREN

### AN ANALYSIS OF SIX HUNDRED AND SIXTY TUMOURS

G. H. COORAY AND RANEE PERERA

*From the Department of Pathology, University of Ceylon, Colombo*

Received for publication November 4, 1965.

MANY children in Ceylon suffered from and died of infectious diseases before antibiotics were introduced. The control of these has altered the pattern of childrens' diseases in this country to a certain extent. During the last few years neoplastic disease has played no small a part in increasing the death rate of infants and children. Thus in a study made by De Silva (1953) malignancy (including leukaemias) caused the second highest fatality rate among medical admissions to the Lady Ridgeway Hospital for children in Colombo, coming second only to that of prematurity. Among the surgical admissions, tumours (excluding leukaemias) were sixth in frequency.

Although neoplasms have assumed this degree of importance in contributing to morbidity and mortality in infants and children in this country, no attempts have hitherto been made to study the tumours occurring during this age period and no paper has been published to date dealing with this special local problem. We were therefore prompted to make an analysis of all tumours sent to our department from the Lady Ridgeway Hospital, Colombo, as well as from other smaller childrens' hospitals in Ceylon. (Our department being a central laboratory receives specimens from all over Ceylon and our findings may be regarded as being a fairly accurate picture of this problem.) We have also taken this opportunity to compare some of our findings with those of other workers in India as well as in the West in order to ascertain whether any differences exist in the pattern of neoplastic disease between Ceylon and other countries.

Our study is based on specimens of tumours of infants and children under the age of 15 years which were received by our department, during the seven year period 1957–63. In some cases whole tumours removed by surgery were examined while in others, the material examined was a surgical biopsy. Only a very few specimens were obtained at autopsy. Leukaemias are not included in this series as such cases are examined by hospital pathologists.

During this period, 660 such specimens were received and of these, the innocent tumours (excluding those of neural origin) amounted to 400 (60·6%).

Malignant tumours (excluding those of neural origin) amounted to 164 (24·9%) and 96 (14·5%) were derived from neural tissue, a category which contained both innocent and malignant tumours (Table I).

TABLE I.—*Types of Tumours*

| | Number of Tumours | % |
|---|---|---|
| Innocent tumours | 400 | 60·6 |
| Malignant tumours | 164 | 24·9 |
| Neural tumours | 96 | 14·5 |
| Total | 660 | |

### (1) *Innocent Tumours*

These have been classified into the following categories as shown in Table II.

TABLE II.—*Classification of Innocent Tumours*

| | Number of Tumours | % |
|---|---|---|
| Mesenchymal | 231 | 57·8 |
| Epithelial | 157 | 39·2 |
| *Special | 12 | 3 |
| Total | 400 | |

* In classifying certain tumours as " Special " we have followed Willis (1960a).

Table III is a histological classification of the mesenchymal tumours.

TABLE III.—*Histological Classification of Mesenchymal Tumours*

| | Number | % |
|---|---|---|
| Haemangioma | 115 | 50·0 |
| Lymphangioma | 35 | 15·1 |
| Lipoma | 25 | 10·8 |
| Fibroma | 13 | 5·6 |
| Osteoma and chondroma | 12 | 5·2 |
| Synovioma (ganglion) | 10 | 4·3 |
| Meningioma | 6 | 2·6 |
| Osteoclastoma | 4 | 1·7 |
| Bone cyst | 4 | 1·7 |
| Xanthogranuloma | 3 | 1·3 |
| Leiomyoma | 2 | 0·9 |
| Histiocytoma | 2 | 0·9 |
| Total | 231 | |

More than half the mesenchymal tumours belonged to the group of vascular tumours, namely haemangiomas and lymphangiomas. Twenty-five were fatty tumours. A noteworthy feature was the occurrence of 6 meningiomas and 4 osteoclastomas—3 occurring in the jaw and 1 in the tibia. There were also 10 synoviomas (ganglion), all excepting 3 being situated in the region of the wrist. Out of the 3 remaining synoviomas, 2 occurred in the foot and 1 in the popliteal fossa.

Table IV is a histological classification of the epithelial tumours.

Amongst the epithelial tumours, cysts contributed to more than two-thirds, dermoid cysts amounting to 49 and other kinds of cysts amounting to 41. Forty-two of the dermoid cysts arose in the skin and 4 within the cranium (cholesteatomas). The other kinds of cysts had a fairly wide field of origin and included 8 sebaceous cysts, 7 lung cysts, 6 thyroglossal cysts, 6 ranulas, 3 mucus retention cysts, 3 branchial cysts, 2 dentigerous cysts, 2 corpus luteal cysts, 2 arachnoidal cysts and 1 cyst in the parotid and 1 within the mesentery.

TABLE IV.—*Histological Classification of Epithelial Tumours*

| | Number | % |
|---|---|---|
| Dermoid cysts | 49 | 31·2 |
| Other cysts | 41 | 26·1 |
| Papillomas (skin warts) | 24 | 15·2 |
| Gastro-intestinal polyps | 9 | 5·7 |
| Fibroadenoma breast | 6 | 3·8 |
| Pleomorphic adenoma (salivary gland) | 6 | 3·8 |
| Umbilical adenoma | 6 | 3·8 |
| Ameloblastomas | 4 | 2·6 |
| Thyroid adenoma | 3 | 1·9 |
| Hepatoma | 2 | 1·3 |
| Sebaceous adenoma | 2 | 1·3 |
| Sweat gland adenoma | 1 | 0·6 |
| Cystadenoma parotid | 1 | 0·6 |
| Cystadenoma ovary | 1 | 0·6 |
| Parathyroid adenoma | 1 | 0·6 |
| Adereno-cortical adenoma | 1 | 0·6 |
| Total | 157 | |

Out of the 9 polyps in the gastro-intestinal tract, 7 were from the rectum and 1 each from the colon and small intestine.

Fibroadenomas of the breast, pleomorphic adenomas of the salivary glands and thyroid adenomas occurred between the ages of 12 and 14 years except for 1 adenoma of the thyroid which occurred in a girl of 4 years.

It was noted that all the breast and thyroid tumours occurred in females, while the salivary gland tumours occurred in 4 males and 2 females.

Of the 4 ameloblastomas, 3 occurred in females and the ages of these were 13, 9, 5 years and 2 months respectively. The 2-month-old female had a melanotic adamantinoma fully reported elsewhere (Panabokke and Eaton, 1964).

The parathyroid adenoma occurred in a 13 year old female who suffered from multiple spontaneous fractures, deformities of bone and inability to walk for 7 months.

The adrenal cortical adenoma occurred without any of the signs of Cushing's disease.

The " special " tumours consisted of 8 benign melanomas all occurring in the skin. The ages ranged from 7 months to 14 years with an average of 5·5 years and a sex ratio of 6 males to 2 females. There were also 3 benign teratomas (dermoid cysts of the ovary) which occurred at ages 14, 11 and 5 years and 1 chorio-adenoma (hydatidiform mole) which occurred in a Mohammedan girl of 14 years at her first pregnancy.

### (2) *Malignant Tumours*

The 164 malignant tumours consisted of 98 (59·7%) tumours of mesenchymal origin excluding leukaemias, 18 (11%) of epithelial origin and 48 (29·3%) "special" tumours. These "special" tumours consisted of 25 embryomas, 22 teratomas and 1 chordoma. (Table V).

*Malignant mesenchymal tumours*

In the group of tumours of lymphoreticular tissues, 23 (nearly half) were cases of Hodgkin's disease with a male to female ratio of 17 : 6. The ages ranged from 1½ years to 14 years with an average of 8·1 years. Sixteen of the 23 cases arose in

TABLE V.—*Classification of Malignant Tumours*

| | | | | |
|---|---|---|---|---|
| *Mesenchymal Tumours* 98 (59·7%) | Tumours of lymphoreticular tissues | | 48 | 29·3% |
| | Hodgkin's lymphoma | 23 | | |
| | Reticulum cell sarcoma | 13 | | |
| | Lymphosarcoma | 12 | | |
| | Bone tumours | | 22 | 13·4% |
| | Osteosarcoma | 8 | | |
| | Ewing's sarcoma | 8 | | |
| | Chondrosarcoma | 4 | | |
| | Plasmacytoma | 2 | | |
| | Soft tissue tumours | | 28 | 17·1% |
| | Malignant synovioma | 9 | | |
| | Fibrosarcoma | 2 | | |
| | Myosarcoma | 2 | | |
| | Liposarcoma | 1 | | |
| | Vascular tumours | 3 | | |
| | Mesenchymoma | 2 | | |
| | Meningioma | 1 | | |
| | Unclassified | 8 | | |
| *Epithelial tumours* 18 (11·0%) | Skin epitheliomas | | 9 | 5·5% |
| | Ovary | | 4 | 2·4% |
| | Dysgerminomas | 2 | | |
| | Mesonephroma | 1 | | |
| | Malignant papillary cystadenoma | 1 | | |
| | Parotid—Muco-epidermoid carcinoma | | 2 | 1·2% |
| | Kidney—Hypernephroma | | 1 | 0·6% |
| | Rectum—Adenocarcinoma | | 1 | 0·6% |
| | Thyroid carcinoma | | 1 | 0·6% |
| "*Special*" *tumours* 48 (29·3%) | Embryomas | | 25 | 15·2% |
| | Teratomas | | 22 | 13·4% |
| | Chordoma | | 1 | 0·6% |
| | Total | | 164 | |

the cervical glands. The other infrequent sites were axilla, inguinal glands and skin.

The other half of the tumours in this group consisted of 13 reticulum cell sarcomas and 12 lymphosarcomas. The ages of patients with reticulum cell sarcomas ranged from 3 to 14 years with an average of 9·2 years, male to female ratio being 7 : 6. Seven of these were cervical in origin. An unusual site was the presence of 2 tumours in the spinal cord.

The ages of those with lymphosarcomas ranged from 4 to 14 years with an average of 10 years. The sex ratio was 8 males to 4 females. Seven out of the 12 were in the cervical region, 2 in the axilla, 2 in the intestines and 1 in the region of the jaw.

The fifty remaining mesenchymal neoplasms originated in bones and soft tissues. Of the bone tumours, osteosarcomas and Ewing's sarcomas appeared to be the most frequent. All the osteosarcomas occurred in the long bones, with an average age incidence of 12 years and male to female ratio of 7 : 1. The Ewing's sarcoma occurred at an average age of 11 years, the sex ratio being 1 male to 7 females. Two occurred in the tibia, and 1 each in the humerus, femur, fibula, clavicle, and a rib.

The most frequent soft tissue tumour was the malignant synovioma. The ages of these patients ranged from 10 months to 13 years with an average age incidence of 9·1 years. The sex ratio was 6 males to 3 females.

*Malignant epithelial tumours*

This was a small group of 18 tumours (11% of the malignant tumours). The largest number (9) arose in the skin and of these 4 were situated in the neck and were branchiogenic in origin. Two arose from sebaceous glands and the origin of the remaining 3 could not be determined as they were very anaplastic. The ages of the patients with these tumours ranged from 1 to 14 years with an average of 9·4 years. The sex ratio was 6 males to 2 females.

The next frequent site was the ovary which gave origin to 4 tumours. Two of these were dysgerminomas occurring at 14 and 10 years respectively, one a mesonephroma at 2 years and 9 months and the other a papillary cystadenoma at 7 years.

Both parotid tumours, in a 10 year old boy and 6 year old girl, were mucoepidermoid carcinomas.

The thyroid, kidney, rectum and pelvis each gave rise to a tumour at ages, 8, 7, 9 and 2 years respectively. The thyroid carcinoma and the hypernephroma occurred in males, while the rectal papillary adenocarcinoma and the pelvic tumour occurred in females.

"*Special tumours* (*Table V*)

The embryomas consisted of 21 nephroblastomas (Wilms' tumours) three hepatoblastomas and 1 malignant embryonic abdominal tumour. Table VI gives the age frequencies of the nephroblastomas.

TABLE VI.—*Age Frequencies of Nephroblastomas*

| | |
|---|---|
| 1 year | 1 |
| 1½ years | 3 |
| 2 ,, | 6 |
| 3 ,, | 3 |
| 4 ,, | 3 |
| 4½ ,, | 1 |
| 5 ,, | 2 |
| 7 ,, | 2 |
| Total | 21 |

It will be seen that the largest number of cases (16) occurred between the ages of 1 and 4 years. The sex ratio of the Wilms' tumours was 13 males to 8 females.

Two of the three hepatoblastomas occurred in males. The ages of these were 2 years, 1½ years and 10 months respectively. There was 1 malignant embryonic abdominal tumour in an 8-month-old girl.

The sites of the teratomas is given in Table VII.

TABLE VII.—*Site Incidence of Teratomas*

| | |
|---|---|
| Sacrococcygeal | 9 |
| Testis | 4 |
| Retroperitoneal | 3 |
| Stomach | 2 |
| Brain | 1 |
| Thyroid | 1 |
| Skull | 1 |
| Ovary | 1 |
| Total | 22 |

The largest number (9) occurred in the sacrococcygeal region and 4 occurred in the testis. Two teratomas occurred in an unusual site, namely the stomach. These two cases have been fully reported elsewhere (Cooray and Jayaratne, 1959 ; Paul, Cooray and Wickramasinghe, 1962). The sex ratio was 7 males to 15 females and the youngest child was 1 day old with a sacrococcygeal teratoma and the oldest 12 years with a malignant ovarian teratoma. Eight cases (i.e. more than one third) occurred in children under 2 years.

The only chordoma occurred in a female child 12 years old.

### (3) *Neural Tumours*

There were 96 tumours derived from neural tissue which accounted for 14·5% of the total number of tumours. Table VIII gives the types of neural tumours.

TABLE VIII.—*Classification and Sites of Neural Tumours*

| | Brain and spinal cord | Other sites | Total | % |
|---|---|---|---|---|
| Gliomas | 29 | — | 29 | 30·2 |
| Neurofibromas | 4 | 28 | 32 | 33·4 |
| Retinoblastomas | — | 24 | 24 | 25·0 |
| Medullo-epithelioma | — | 1 | 1 | 1·0 |
| Neuroblastomas | — | 9 | 9 | 9·4 |
| Phaeochromocytoma | — | 1 | 1 | 1·0 |
| Total | 33 | 63 | 96 | 100 |

About a third (33) arose from the brain and spinal cord and the remainder (63) were from other sites. All the 29 gliomas arose from the brain and of the neurofibromas 26 were dermal in origin.

Of the retinoblastomas, 15 occurred in females and 9 in males, and the only medullo-epithelioma occurred in a 4 month old boy who was born with a squint. The highest age for the retinoblastomas was 7 years and the lowest 1 year with an average age of 3·1 years. None of these cases gave a family history.

Of the 9 neuroblastomas, 8 occurred in the adrenals and 1 in the cervical sympathetic. All excepting 1 were females. The youngest was a 1 year old girl and the oldest a 7 year old boy, the average age incidence being 4·0 years.

The only phaeochromocytoma was an autopsy specimen from a 13 year old girl who developed sudden abdominal pain with collapse and died within 24 hours. There was no opportunity to ascertain whether this tumour produced any endocrine effects.

Table IX gives the various histological types as well as the sex incidence of the gliomas.

TABLE IX.—*Sex Incidence of the Various Types of Gliomas*

| | Males | Females | Total |
|---|---|---|---|
| Astrocytoma | 4 | 4 | 8 |
| Glioblastoma multiforme | 3 | 1 | 4 |
| Medulloblastoma | 4 | 5 | 9 |
| Ependymoma | 4 | 2 | 6 |
| Oligodendroglioma | — | 1 | 1 |
| Colloid cyst | 1 | — | 1 |
| Total | 16 | 13 | 29 |

It will be noted that the most frequent histological types were the medulloblastomas, astrocytomas and ependymomas. There was no significant difference in the sex incidence.

The average ages for the different histological types of gliomas were as follows : astrocytoma 8·5 years, glioblastoma multiforme 9·5 years, medulloblastoma 7 years, ependymoma 6·1 years and oligodendroglioma 6 years.

*Age frequency.*—Fig. 1 represents the frequency of the neural tumours, malignant mesenchymal and malignant " special " tumours as well as the carcinomas

FIG. 1.—Age frequency of various groups of tumours in children.

— — — — Malignant mesenchymal tumours
—·—·— Neural tumours
———— Malignant special tumours
- - - - - - - Malignant epithelial tumours occurring at different age periods. It will be seen that neural and " special " tumours occur more frequently in the 0–4 age group due to the high prevalence rate of retinoblastomas, nephroblastomas and teratomas during this age period. The slope of the graph for neural tumours, however, is not so steep as that of the " special " tumours, because in the former case other neural tumours such as the medulloblastomas, astrocytomas and neurofibromas occur at later ages, whereas in the case of the latter, nephroblastomas and teratomas do not appear at later age groups. The mesenchymal tumours appear to be less frequent during the early age periods but tend to rise with increase of age. The carcinomas are few at all ages but there is a tendency for their frequency to rise with age.

### DISCUSSION

The population of Ceylon, which is composed of several races, according to the last Census Report (1963) was 10,624,507. 40·7% of this population was between 1 to 14 years.

That neoplastic disease plays an important role in contributing to morbidity during these age periods is shown by the fact that during a seven year period 660 tumours (innocent and malignant) have been examined by one pathology department (average of about 94 per year). The actual incidence of tumours in this age group is bound to be much higher because quite a large number of children suffering from neoplastic disease in this country either die at home or are referred to practitioners of the indigenous system of medicine.

Lawrence and Doulan (1952) from the Indiana University Medical Centre collected 1409 innocent and malignant tumours during a period of 27 years (average of about 52 per year). The significance of tumours in infancy and childhood in comparison with other diseases, as a cause of death, has been stressed by Dargeon (1940) in America, Sirsat (1953) in India, O'Conor and Davies (1960) in East Africa, Muir (1961) in Singapore and Steward (1964) in Britain.

It will be seen from the foregoing observations that almost all types of tumours which occur in adults arise in infants and children. Certain tumours like the medulloblastoma, neuroblastoma Wilms' tumour and retinoblastoma are, however, peculiar to children, while the carcinomas are somewhat rare. Another fact which emerges from this study is that every type of tumour met with in Western Countries occurs in infants and children of Ceylon.

The pattern of neoplastic disease in Ceylon does not appear to be dissimilar to that of other countries. For instance, in the case of innocent tumours nearly 60% arose in mesenchymal tissues and 115 or 50% were haemangiomas. Lawrence and Doulan (1952) made a similar observation in respect of innocent tumours examined at the Indiana University. Although the epithelial tumours comprised just over a third of the number of innocent tumours a perusal of Table IV shows that almost every type was represented. Cysts of new formation were the most prominent, the majority arising in the skin while others had a wide field of origin (*vide supra*). The melanoma was another tumour which frequently arose in the skin. Older children developed fibroadenoma of the breast. A noteworthy feature was the occurrence of a chorio-adenoma in a young girl of 14 years. (Early marriage is not uncommon in this country and the chorio-adenoma occurs more frequently in Ceylon than in other countries (Cooray, 1954)).

In the malignant group of tumours those arising from mesenchymal tissues again exceeded any other kind of tumour (Table V). If the number of leukaemias about which we have no knowledge (as such cases are referred to hospital pathologists) is added to mesenchymal tumours, as is done by other workers in this field (e.g. Steward, 1964; O'Conor and Davies, 1960; Campbell *et al.*, 1961) the frequency of all tumours of mesenchymal origin will indeed be very high. In this respect the pattern of malignant tumour growth in infancy and childhood is not different from that of other countries (Dargeon, 1940a; Sirsat, 1953; Muir, 1961; Steward, 1964).

Among the mesenchymal tumours 29·3% were tumours of lymphoreticular tissues (excluding leukaemias). The most frequent histological type met with was the Hodgkin's lymphoma, the other types, namely the reticulum cell sarcoma and lymphosarcoma, being less common.

Davies (1964), however, has reported a higher frequency of lymphosarcomatous tumours in Uganda on account of the very high incidence of Burkitt's tumour, which is not met with in Ceylon.

Next in frequency to tumours of the lymphoreticular tissues were the soft tissue tumours which amounted to 17·1%. Nearly one-third of these were malignant synoviomas which is considered to be a rare condition in childhood (Anderson and Perrin, 1959).

Malignant neoplasms of bone amounted to 13·4%, osteosarcomas and Ewing's sarcomas being the most frequent types met with in Ceylon. Our findings are in agreement with Dargeon (1940a) who reported that bone tumours formed 11·6% of the malignant neoplasms. Contrary to reports from other countries (Geschickter and Copeland, 1936 ; Kirkpatrick, 1959), Ewing's sarcoma occurred much more frequently in the females.

In considering the embryomas, teratomas and chordomas as "special" tumours we have followed Willis (1960a). The embryoma was the type most frequently encountered amounting to 15·2% of the malignant tumours. Of these, 21 were nephroblastomas (Wilms' tumour). Although this tumour was met with less frequently in the series reported by Steward (1964), Dargeon (1940a) regards this as one of the most frequent types seen in infancy. The age frequency was not different from other reported series. Although Wilms (1899) in his monograph and in subsequent series of case reports recorded an equal sex incidence, the present series showed that males were more frequently affected than females.

The teratomas constituted a numerically important group of tumours and their malignant behaviour made them a serious problem. The sex frequency showed a marked female predominance. The age frequency and the site incidence were not different from other reported series (Willis, 1960b).

Malignant epithelial tumours were seen but rarely—only 11% in this series being carcinomas. The frequency of malignant epithelial tumours is higher than in Bombay where Desai and Bhansali (1964) records an incidence of 6%. However, the observation that carcinomas in Ceylonese infants and children are rare in comparison with other types of malignant tumours is in agreement with the findings of workers in western countries (Pack and Lefevre, 1930 ; Gaisford, 1949). A noteworthy feature was the occurrence of 9 skin carcinomas, 4 being considered branchiogenic in origin.

Although tumours derived from neural tissues amounted to only 14·5% of the total number of tumours, a much higher frequency of such tumours is noticeable if its proportion among only the malignant tumours is calculated. On this basis Campbell *et al.* (1961) recorded 143 neural tumours out of a total of 470 (30·4%) and Steward (1964) 245 out of 890 (27·5%). In our series there were 63 neural tumours (excluding neurofibromas) out of 228 malignant tumours (27·6%). It will thus be seen that such tumours occur with the same frequency as in western countries and in certain parts of India (Desai and Bhansali, 1964).

A very high proportion of these tumours arose from the brain, the retina and adrenal medulla while a large number arose from cutaneous nerves. These were all solitary neurofibromas and no cases of multiple neurofibromatosis was encountered in this series. Of the glial tumours the medulloblastomas, astrocytomas and ependymomas were the most frequent histological types. The average age frequency of medulloblastomas, namely 7 years, appears to be higher than in other countries. In none of the retinoblastomas was there a familial history, an observation which is in agreement with that of Willis (1958) who states that most cases of retinoblastoma are sporadic, no siblings or other near relations being affected. Contrary to reports that they show any predilection to sex (McGavic, 1959; Dargeon, 1960) there was a preponderance of females affected in the present series. The neuroblastomas also showed a predilection for the male sex although it is stated that the sex incidence is equal (Bodian, 1959). Regarding anatomical localisation, a perusal of the tables reveals that the sites from which malignant tumours most frequently arose were lymph glands (48 cases), brain (33 cases), soft somatic tissues (28 cases), eye (25 cases), bones (22 cases), kidney (21 cases), adrenal glands (9 cases) and sacrococcygeal (9 cases). The exact proportion of tumours in any one organ is difficult to determine. Dargeon (1940b) records a series of observations regarding anatomical distribution given by various authors. Our results show minor variations on these figures but they are in close agreement with the site incidence recorded by Sirsat in Bombay (1958).

### SUMMARY AND CONCLUSIONS

1. Six hundred and sixty neoplasms from Ceylonese infants and children under 15 years of age have been examined at the Department of Pathology, University of Ceylon, during the seven year period 1957–1963. Four hundred (60·6%) of these were innocent tumours, 164 (24·9%) were malignant tumours and 96 (14·5%) were tumours of neural tissue origin. (In this last category were both innocent and malignant tumours.)

2. Neoplasms derived from mesenchyme constituted a very large proportion of the innocent tumours (57·8%), while 39·2% were tumours of epithelial origin. Among the " special " tumours, which constituted only 3% of the innocent tumours, the innocent melanoma was the most frequent. An unusual feature was the occurrence of a chorio-adenoma (hydatidiform mole) in a girl of 14 years.

3. Although innocent epithelial tumours were fewer in number almost every type was represented, a very large proportion being cysts of new formation.

4. Mesenchymal tumours (excluding leukaemias) constituted a large proportion (59·7%) of the malignant tumours too. Carcinomas were very rare (11%) and " special " tumours, chiefly embryomas and teratomas, amounted to 29·3% of the malignant tumours. Tumours of neural origin amounted to 27·6% of the malignant tumours.

5. In the mesenchymal group the largest number (29·3%) originated in lymphoreticular tissues. Soft tissues gave origin to 17·1% and bone to 13·4%. In the " special " group of tumours the embryomas and teratomas were the neoplasms most frequently met with and in the former category nephroblastoma (Wilms' tumour) was the most common.

6. The most frequent tumours of neural origin were the intracranial gliomas, the cutaneous neurofibromas, the retinoblastomas. Of the gliomas the most frequent histological types were the medulloblastomas, astrocytomas and ependymomas.

7. The anatomical sites most frequently affected were the lymph glands, brain, soft somatic tissues, eye, bones, kidney, adrenal glands, sacrococcygeal region.

8. The retinoblastomas, nephroblastomas (Wilms' tumour) and teratomas occurred in the age group 0–4, and the medulloblastomas in the age group 5–9 years. The mesenchymal tumours were uncommon in the early age group but were more frequently seen between 10 and 14 years. The neurofibromas occurred in all age groups while the carcinomas which were very few occurred at all ages.

9. In general, the pattern of neoplastic disease in infants and children follows that observed in western countries and certain parts of India. There are, however, minor variations which have been indicated in this paper.

### REFERENCES

Anderson, D. H. and Perrin, E. V.—(1959) *Pediat. Clins. N. Am.*, **6**, 548.

Bodian, M.—(1959) *Pediat. Clins. N. Am.*, **6** 450.

Campbell, A. C. P., Gaisford, W. , Paterson, E. and Steward, J. J.—(1961) *Br. med. J.*, i, 448.

Census Report (Ceylon)—(1963) Department of Census and Statistics, Ceylon.

Cooray, G. H.—(1954) *Acta Un. int. Cancr.*, **10**, 34.

Cooray, G. H. and Jayaratne, S.—(1959) *Archs Path.*, **67**, 383.

Dargeon, H. W.—(1960) ' Tumours of Childhood'. New York (Paul B. Hoeber), p. 99.

Dargeon, H. W.—(1940a) ' Cancer in childhood '. London (Kimpton), p. 15.

Dargeon, H. W.—(1940b) ' Cancer in childhood '. London (Kimpton), p. 26.

Davies, J. N. P.—(1964) ' Lymphomas and Leukaemias in Uganda Africans ', Symposium on " Tumours in Africa ", in Paris 1963. New York, pp. 67–74.

Desai, P. B. and Bhansali, S. K.—(1964) *Indian J. Cancer*, **1**, 1.

De Silva, C. C.—(1953) *Acta paediat., Stockh.*, **42**, 453.

Gaisford, W. F.—(1949) *Archs Dis. Childh.*, **24**, 1.

Geschickter, C. F. and Copeland, M. M.—(1936) ' Tumors of Bone ', N. York. (Amer. J. Cancer), p. 381.

Kirkpatrick, J. A.—(1959) *Pediat. Clins N. Am.*, **6**, 557.

Lawrence, E. A. and Doulan, E. J.—(1952) *Cancer Res.*, **12**, 900.

McGavic, J. S.—(1959) *Pediat. Clins N. Am.*, **6**, 559.

Muir, C. S.—(1961) *Cancer*, **14**, 534.

O'Connor, G. T. and Davies, J. N. P.—(1960) *J. Pediat.*, **56**, 526.

Pack, G. T. and LeFevre, R. G.—(1930) *J. Cancer Res.*, **14**, 167.

Panabokke, R. G. and Eaton, H. L.—(1964) *Ceylon med. J.*, **9**, 63.

Paul, M., Cooray, G. H. and Wickramasinghe, S. Y. D. C.—(1962) *Br. J. Surg.*, **50**, 220.

Sirsat, M. V.—(1953) *Indian J. med. Res.*, **41**, 277.

Sirsat, M. V.—(1958) *Indian J. med. Res.*, **46**, 293.

Steward, J. K.—(1964) *J. clin. Path.*, **17**, 407.

Willis, R. A.—(1958) ' The Borderland of Embryology and Pathology '. London (Butterworth & Co.), p. 417.

Willis, R. A.—(1960a) ' Pathology of Tumours ', 3rd edition. London (Butterworth & Co.), p. 18.

Willis, R. A.—(1960b) ' Pathology of Tumours ', 3rd edition. London (Butterworth & Co.), p. 944.

Wilms, M.—(1899) Cited by Cresson, S. L. and Pilling, G. P.—(1959) *Pediat. Clins N. Am.*, **6**, 473.